# Supplementary material for: Developing and Piloting a Novel Ranking System to Assess Popular Dietary Patterns and Healthy Eating Principles
Source: Nutrients. 2022 Aug 19;14(16):3414. doi: 10.3390/nu14163414 (PMC9415867; doi:10.3390/nu14163414)
Supplement: Supplementary file 1 [file nutrients-14-03414-s001.zip › nutrients-1858892-supplementary.pdf]

Developing and piloting a novel ranking system to assess popular dietary patterns and healthy eating principles

Ella L Bracci [1*], Rachel Milte[2,] Jennifer B Keogh [1] and Karen J Murphy[1]

Supplementary information

Table S1: Nutrition Profile of diets

Table S2: Ranking System Outline & Categories

Table S1. Summary of nutrition profile of popular weight loss diets and healthy eating principles

Nutrients	Keto	Paleo	IF	8WW	Optifast	MedDiet WL	AGHE WL
Energy (avMJ/day)	8.9	6.7	6.5	7.3	5.0	6.9	6.1
%en from protein	22	23	24	56	26	20	20
%en from CHO	6	23	36	11	48	31	47
%en from Fat	70	51	35	29	19	40	28
%en MUFA	27	26	16	10	9	22	12
%en PUFA	10	7	6	3	3	8	5
%en SFA	29	18	10	11	4	8	9
%en alcohol	nil	0.1	0.1	nil	0.6	4.0	0.3
Fibre (g/day)	20	25	34	21	43	29	27
Vit C (mg/day)	90	242	166	193	630	215	135
Vit E(mg/day)	29	26	15	14	21	23	11
Vit A (µg/day)	831	1363	983	992	2095	1005	1246
Folate (µg/day)	355	283	446	483	943	451	555
VitB12 (µg/day)	5.4	3.0	4.0	15	3.0	3.4	3.9
Vit B1 (mg/day)	1.0	0.8	1.2	0.9	2.0	1.1	1.0
Niacin (mg/day)	50	40	34	111	40	35	30
Magnesium (mg/day)	398	350	446	448	451	339	355
Iodine (µg/day)	98	54	142	123	138	112	152
Phosphorous (mg/day)	1926	1242	1672	2647	1457	1350	1299
Selenium (µg/day)	103	78.9	86.1	228	69.9	71.9	75.5
Sodium (mg/day)	2922	1820	3272	1786	3903	1214	1727
Potassium (mg/day)	2802	3310	3730	5261	5776	3716	3059
Calcium (mg/day)	1249	366	1368	898	1203	782	953
Iron (mg/day)	12	11	12	16	19	11	8.7
Zinc	13	11	10	24	13	9.7	9.1

**Serves/Day**							
Fruit	0.2	1.4	0.9	0.2	2.3	1.0	2.0
Vegetables	2.5	5.9	5.9	6.5	12	8.3	4.2
Breads and cereals	0.5	0.8	4.5	0.4	0.7	3.2	4.8
Dairy foods	2.3	nil	2.8	1.9	1.2	1.3	0.9
Meat/meat alt.	6.1	5.0	2.4	9.1	0.6	2.8	2.0
Discretionary	3.3	2.1	1.1	0.1	0,3	1.3	0.9

CHO= carbohydrate, PRO= protein, %en = percentage of energy, MUFA= monounsaturated fat, PUFA= polyunsaturated fat, SFA= saturated fat, IF= intermittent fasting, 8WW = 8 Weeks to Wow

Table S2 Ranking System Outline & Categories

Category: Dietary Quality	Score (points)	Comments
i.Meal plan/diet Accounts for gender?		
Yes	10	
No	0	
Food Groups & Serve Sizes (based on Foodworks data – averages)		
a) Fruit intake		
0 serves/day	0	
1 serve/day	10	
2 serves/day (100%)	20	
b) Vegetable intake		
0 serves/day	0	
1 serve/day	10	
2 serves/day	20	
3 serves/day	30	
4 serves/day	40	
5 serves/day (100% F)	50	
6 serves/day (100% M)	50	
c) Dairy & alternatives*		

0 serves/day<br>1 serve/day<br>2 serves/day (100% F)<br>2.5 serves/day (100% M)	0<br>10<br>20<br>20	
d) Breads, cereals & grains<br>0 serves/day<br>1 serve/day<br>2 serves/day<br>3 serves/day (50%)<br>4 serves/day<br>5 serves/day<br>6 serves/day (100% F + M)	 0<br>10<br>20<br>30<br>40<br>50<br>60	
e) Lean meats, poultry & alternatives*<br>0 serves<br>1 serve/day<br>2 serves/day<br>2.5 serves/day (100% F)<br>3 serves/day (100% M)	 0<br>10<br>20<br>30<br>30	
f) Discretionary<br><2.5 (F)<br><3 (M)<br>>3	 10<br>10<br>0	Manual calculation from Foodworks
i. AMDR Range – in the range Y/N?<br>CHO (45-65%)<br>FAT (20-35%)<br>PRO (15-25%)<br><br>Alcohol (as a beverage)<br>>20g (2x standard drinks)<br><20g	 <br>0 to 10<br>0 to 10<br>0 to 10<br><br><br>-10<br>0	
“Disease prevention”		
g) Limit saturated fat intake<br>(McNaughton) Cholesterol & CHD		

<10% energy<br>10%<br>>10% energy	10<br>0<br>-10		
h) Choose foods low in salt<br>(McNaughton) Hypertension<br><1000mg (AI)<br><2000mg (SDT)<br>>2000mg	 <br>20<br>10<br>-10	SDT 2,000mg/day	
Micronutrients* based on 19-50 y/o			
Fibre<br>Meets Male AI (30g) (100%)<br>Meets Female AI (25g) (100%)<br><AI	 20<br>20<br>0	Males 30g/day Females 25g/day	
Minerals & Trace elements 19-50y/o			
Calcium<br>Meets RDI (100%)<br>Meets EAR (100%)<br><EAR	 20<br>10<br>0	RDI<br>Males 1000mg/day<br>Females 1000mg/day	EAR<br>Males 840mg/day<br>Females 840mg/day
Iron<br>Meets RDI (100%) M<br>Meets RDI (100%) F<br>Meets EAR (100%) M<br>Meets EAR (100%) F<br><EAR	 20<br>20<br>10<br>10<br>0	RDI<br>Males 8mg/day<br>Females 18mg/day	EAR<br>Males 6mg/day<br>Females 8mg/day
Thiamine<br>Meets RDI (100%)<br>Meets EAR (100%)<br><EAR	 20<br>10<br>0	RDI<br>Males 1.2mg/day<br>Females 1.1mg/day	EAR<br>Males 1.0mg/day<br>Females 0.9mg/day
Zinc<br>Meets RDI (100%)<br>Meets EAR (100%)<br><EAR	 20<br>10<br>0	RDI<br>Males 14mg/day<br>Females 8mg/day	EAR<br>Males 12mg/day<br>Females 6.5mg/day
Iodine<br>Meets RDI (100%)<br>Meets EAR (100%)	 20<br>10	RDI<br>Males 150ug/day<br>Females 150ug/Day	EAR<br>Males 100ug/day<br>Females 100ug/day

<EAR	0		
Magnesium<br>Meets RDI (100%)<br>Meets EAR (100%)<br><EAR	 20<br>10<br>0	RDI<br>Males 420mg/day<br>Females320mg/day	EAR<br>Males 350mg/day<br>Females 265mg/day
Phosphorus<br>Meets RDI (100%)<br>Meets EAR (100%)<br><EAR	 20<br>10<br>0	RDI<br>Males 1,000mg<br>Females 1000mg	EAR<br>Males 580 mg/day<br>Females 580mg/day
Selenium<br>Meets RDI (100%)<br>Meets EAR (100%)<br><EAR	 20<br>10<br>0	RDI<br>Males 70ug/day<br>Females 60mg/day	EAR<br>Males 60ug/day<br>Females 50ug/day
Potassium<br>Meets AI (100%)<br><AI	 20<br>0	AI<br>Males 3,800mg/day<br>Females 2,800mg/day	
Vitamins			
Vitamin B12<br>Meets RDI (100%)<br>Meets EAR (100%)<br><EAR	 20<br>10<br>0	RDI<br>Males 2.4ug/day<br>Females 2.4ug/day	EAR<br>Males 2.0ug/day<br>Females 2.0ug/day
Vitamin C<br>Meets RDI (100%)<br>Meets EAR (100%)<br><EAR	 20<br>10<br>0	RDI<br>Males 45mg/day<br>Females 45mg/day	EAR<br>Males 30mg/day<br>Females 30mg/day
Vitamin E *a-tocopherol equiv<br>Meets AI (100%)<br><AI	 20<br>0	AI<br>Males 10mg/day<br>Females 7mg/day	
Folate *DFE<br>Meets RDI (100%)<br>Meets EAR (100%)<br><EAR	 20<br>10<br>0	RDI<br>Males 400ug/day<br>Females 400ug/day	EAR<br>Males 320ug/day<br>Females 320ug/day
Riboflavin<br>Meets RDI (100%)<br>Meets EAR (100%)	 20<br>10	RDI<br>Males 1.3mg/day<br>Females 1.1mg/day	EAR<br>Males 1.1mg/day<br>Females 0.9mg/day

<EAR	0		
Niacin *NE<br>Meets RDI (100%)<br>Meets EAR (100%)<br><EAR	 20<br>10<br>0	RDI<br>Males 16mg/day<br>Females 14mg/day	EAR<br>Males 12mg/day<br>Females 11mg/day
Vitamin A<br>Meets RDI (100%)<br>Meets EAR (100%)<br><EAR	 20<br>10<br>0	RDI<br>Males 900ug/day<br>Females 700ug/day	EAR<br>Males 625ug/day<br>Females 500ug/day
Category –<br>Behaviour Change & Sustainability long-term	Score	Comments	
Support & resources<br>i.e. website, group forum	5		
Meal plan	5		
Strategies/help when eating out, bored/comfort eating	5		
Encourage self-monitoring (counting, recording)	5		
Teaches healthy food principles i.e. label and package reading (self-efficacy)	5		
Promote weight cycling – i.e 8 WW Promoted for the short term	- 5		
Does the diet have restrictions i.e. food groups, foods	-5		
Category – Negative effects	Score	Comments	

Headache<br>Nausea<br>Lethargy<br>Dizziness<br>Diarrhea/constipation	-10<br>-10<br>-10<br>-10<br>-10	Negative cumulative score
Category- Cost of diet	Score	Comments
Food Insecurity<br>(average income = $1,659)<br><br><30% of average income<br>>30% of average income	 <br><br>10<br>-10	
